# Differentiation between Isomeric 4,5-Functionalized 1,2,3-Thiadiazoles and 1,2,3-Triazoles by ESI-HRMS and IR Ion Spectroscopy

**DOI:** 10.3390/molecules28030977

**Published:** 2023-01-18

**Authors:** Dmitrii M. Mazur, Elettra L. Piacentino, Giel Berden, Jos. Oomens, Victor Ryzhov, Vasiliy A. Bakulev, Albert T. Lebedev

**Affiliations:** 1 Organic Chemistry Department, Lomonosov Moscow State University, 119992 Moscow, Russia; 2 Department of Chemistry and Biochemistry, Northern Illinois University, DeKalb, IL 60115, USA; 3 FELIX Laboratory, Institute for Molecules and Materials, Radboud University, Toernooiveld 7, 6525 ED Nijmegen, The Netherlands; 4 Technology of Organic Synthesis Department, Ural Federal University, 620002 Yekaterinburg, Russia

**Keywords:** mass spectrometry, electrospray ionization, 1,2,3-thiadiazoles, 1,2,3-triazoles, isomer identification

## Abstract

A large variety of 1,2,3-thiadiazoles and 1,2,3-triazoles are used extensively in modern pure and applied organic chemistry as important structural blocks of numerous valuable products. Creation of new methods of synthesis of these isomeric compounds requires the development of reliable analytical tools to reveal the structural characteristics of these novel compounds, which are able to distinguish between isomers. Mass spectrometry (MS) is a clear choice for this task due to its selectivity, sensitivity, informational capacity, and reliability. Here, the application of electrospray ionization (ESI) with ion detection in positive and negative modes was demonstrated to be useful in structural studies. Additionally, interconversion of isomeric 4,5-functionalized 1,2,3-triazoles and 1,2,3-thiadiazoles was demonstrated. Application of accurate mass measurements and tandem mass spectrometry in MS2 and MS3 modes indicated the occurrence of gas-phase rearrangement of 1,2,3-triazoles into 1,2,3-thiadiazoles under (+)ESI-MS/MS conditions, independent of the nature of substituents, in line with the reaction in the condensed phase. Infrared multiple photon dissociation (IRMPD) spectroscopy enabled the establishment of structures of some of the most crucial common fragment ions, including [M+H-N_2_]^+^ and [M+H-N_2_-RSO_2_]^+^ species. The (−)ESI-MS/MS experiments were significantly more informative for the sulfonyl alkyl derivatives compared to the sulfonyl aryl ones. However, there was insufficient evidence to confirm the solution-phase transformation of 1,2,3-thiadiazoles into the corresponding 1,2,3-triazoles.

## 1. Introduction

The contemporary chemistry of heterocyclic compounds includes a large variety of organic compounds with 1,2,3-thiadiazole and 1,2,3-triazole moieties. Their derivatives possess a wide range of bioactivity and reactivity [1,2,3,4], so they are commonly used as building blocks in modern organic synthesis [5,6,7], and as target molecules in medicinal [1,8] and analytical [9,10] chemistry. Rapid interconversion between a number of 1,2,3-thiadiazole and 1,2,3-triazole derivatives was shown earlier to be possible even under mild conditions [11,12]. It was also demonstrated that these transformations may be efficiently studied by means of tandem mass spectrometry with electrospray ionization (ESI-MS/MS).

In fact, mass spectrometry has been efficiently used to mimic unimolecular transformations of organic compounds in the gas phase. Similarities between fragmentations in the ion source in various ionization modes and thermolysis, photolysis, and acid- and base-catalyzed reactions in solution have been reported [13,14,15,16,17]. These observations enabled the use of mass spectrometry results to predict the behavior of organic compounds in solution, including direction and yields, as well as to simulate monomolecular reactions of organic compounds in solution. Positive ions appeared to be useful for studying chemical reactions catalyzed by acids [18], and negative ions were applied to study solution reactions triggered by bases [19,20]. Mass spectrometry also enabled differentiation between the linear or cyclic structures of triazoles [19] and thiadiazoles [21]. Moreover, almost all classic organic chemistry rearrangement reactions were found to take place in the ion source of a mass spectrometer [15].

ESI-MS/MS was shown to mimic the interconversion between 1,2,3-thiadiazole and 1,2,3-triazole sulfonylaryl derivatives, dependent on the operation mode [22]. Bearing several polar sites, these compounds tend to rapidly isomerize under mild conditions, which makes ESI-MS/MS the best method for identification of each isomer and for simulation of their mutual rearrangements. The observed fragment ions form distinct fragmentation patterns, which lay down the foundation for the identification of isomeric 1,2,3-thiadiazoles and 1,2,3-triazoles.

**Scheme 1:**
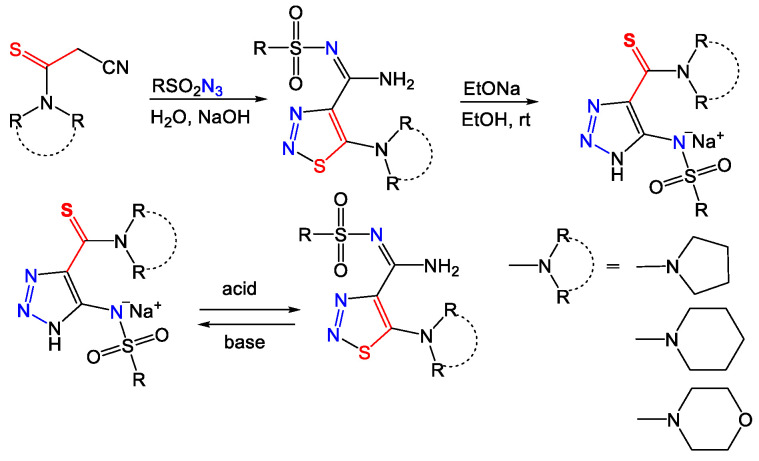
A synthetic route for obtaining 4,5-functionalized 1,2,3-thiadiazoles and 1,2,3-triazoles.

Recently, a more general, effective, and eco-friendly synthesis of variously substituted 1,2,3-thiadiazoles was developed [12]. It was shown that the application of water as a solvent and hydroxide as a base significantly expands the limits of reaction between sulfonyl azides and 2-cyanothioacetamides, providing novel N-sulfonyl- and heteroaryl 5-amino-1,2,3-thiadiazol-4-carbimidamides (Scheme 1). Although the ESI-MS/MS data contain differences between 1,2,3-thiadiazole and 1,2,3-triazole derivatives, which allows tracking of the interconversion and identification of each isomer, there is still a lack of evidence for several proposed fragmentation patterns. At this time, addition of an alternative analytical method in combination with ESI-MS/MS would improve the reliability of the proposed approach for identification of azole isomers. The combination of mass spectrometry with infrared ion-action spectroscopy significantly increases the capabilities of the method for structure elucidation of organic compounds [23,24,25,26]. This technique involves the mass isolation and trapping of the ion of interest, followed by wavenumber-specific photo fragmentation by infrared multiple photon dissociation (IRMPD) using a tunable infrared laser. The molecular vibrational spectra of the ions in the mass spectrometer can in most cases be reliably predicted using common quantum-chemical protocols [27,28,29], allowing for efficient structural characterization, even without standards. The present work demonstrates an ESI-MS/MS study using both positive and negative modes of new N-sulfonylalkyl derivatives of 1,2,3-thiadiazoles and 1,2,3-triazoles with infrared ion spectroscopy (IRIS) for the structure justification of previously proposed fragment ions.

## 2. Results and Discussion

**Scheme 2:**
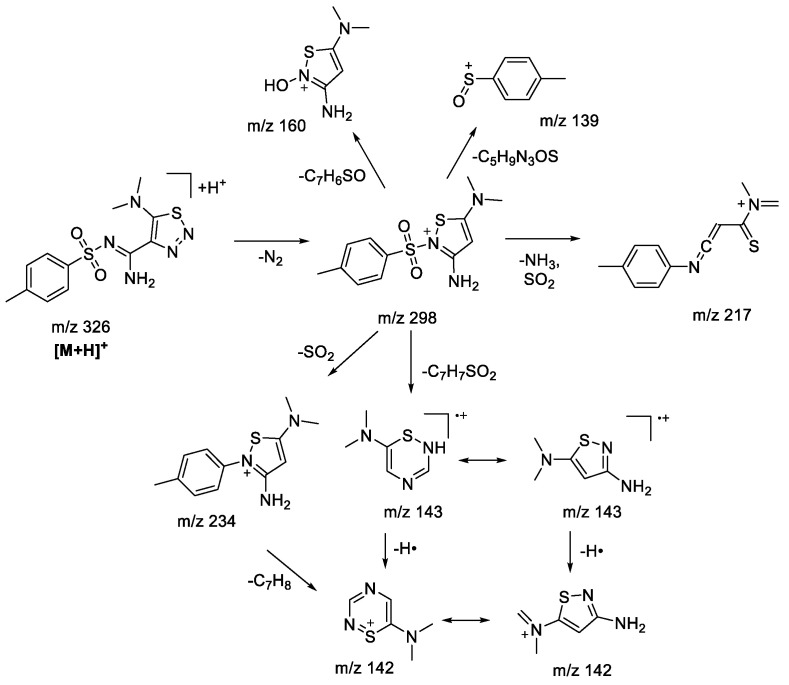
Fragmentation pathways of protonated 1,2,3-thiadiazole **1aS** including the most characteristic ions formed under (+)ESI-MS/MS conditions.

**Scheme 3:**
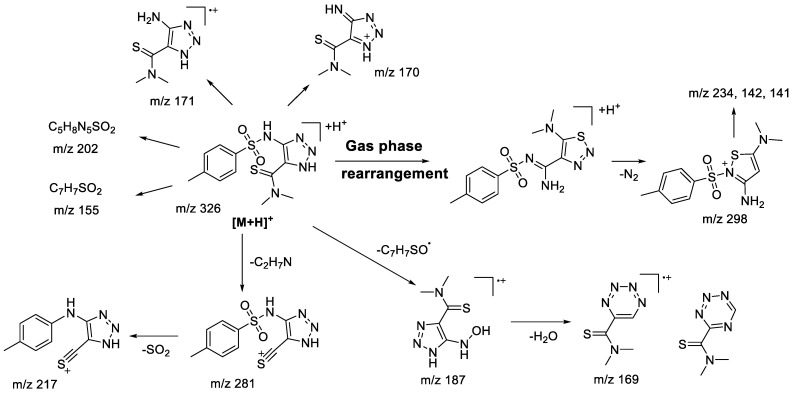
Fragmentation pathways of protonated 1,2,3-triazole **1aN** including the most characteristic ions formed under (+)ESI-MS/MS conditions.

**Scheme 4:**
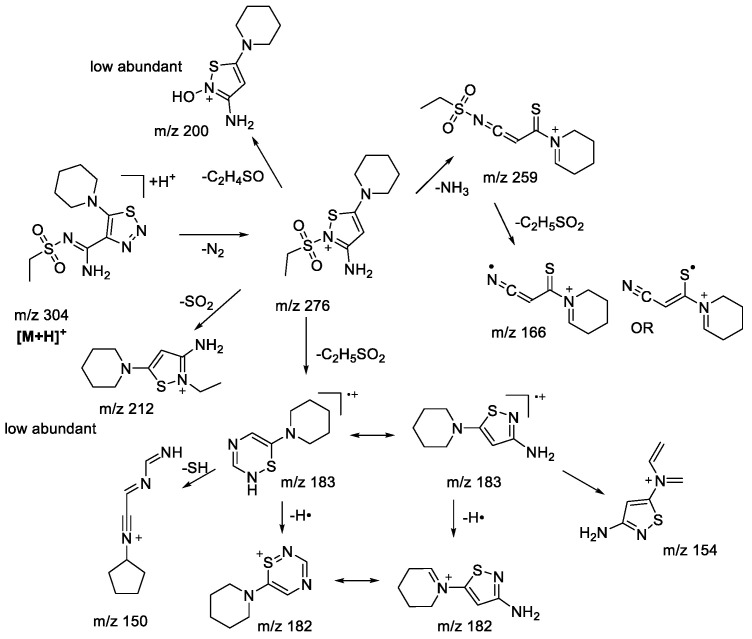
Fragmentation pathways of protonated 1,2,3-thiadiazole **1eS** including the most characteristic ions formed under (+)ESI-MS/MS conditions.

**Scheme 5:**
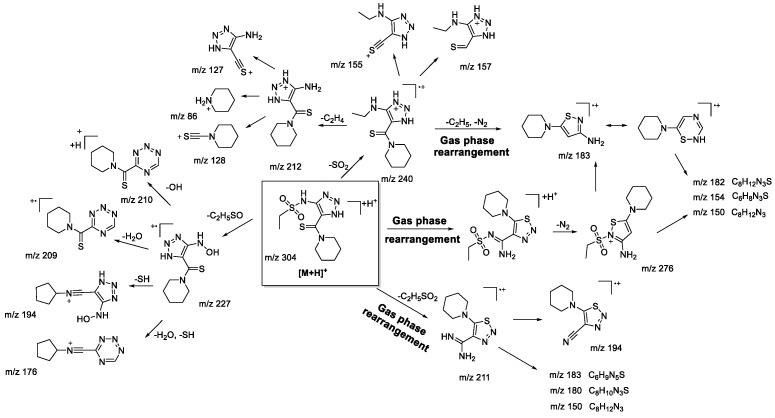
Fragmentation pathways of protonated 1,2,3-thiadiazole **1eN** including the most characteristic ions formed under (+)ESI-MS/MS conditions.

**Scheme 8:**
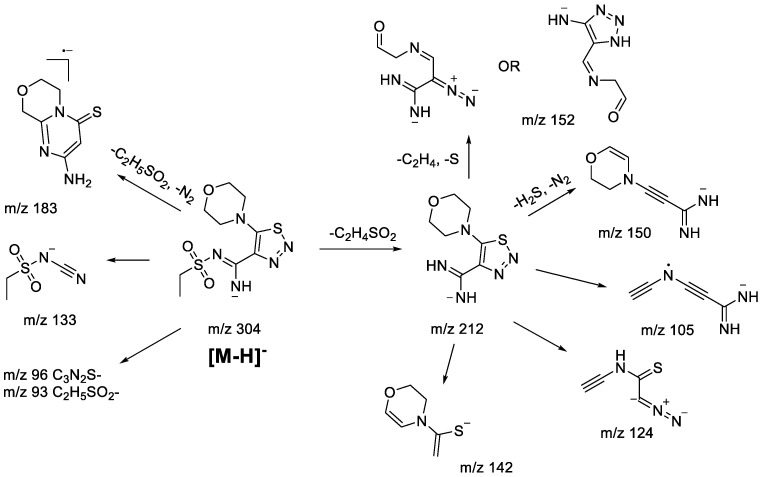
Fragmentation pathways of deprotonated 1,2,3-thiadiazole **1gS** including the most characteristic ions formed under (−)ESI-MS/MS conditions.

**Scheme 9:**
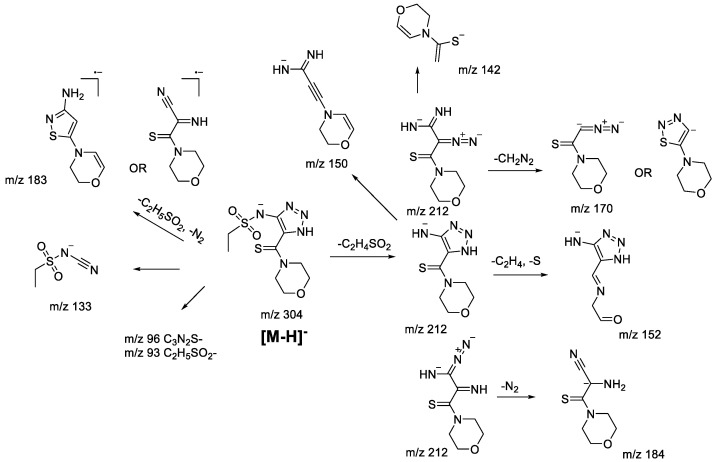
Fragmentation pathways of deprotonated 1,2,3-triazole **1gN** including the most characteristic ions formed under (−)ESI-MS/MS conditions.

**Figure 1:**
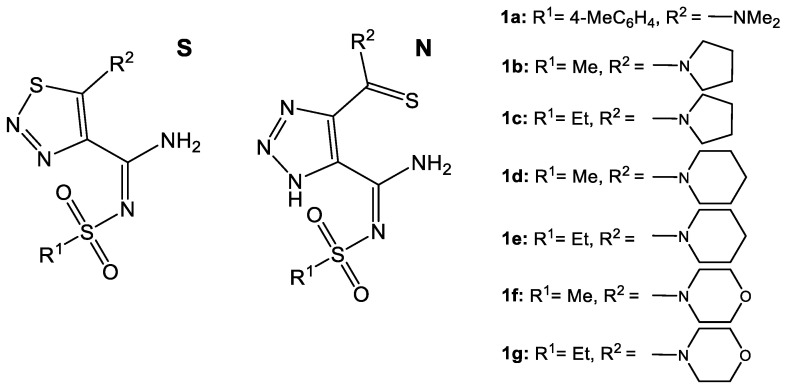
Structures of investigated 4,5-disubstituted 1,2,3-thiadiazoles (S) and 1,2,3-triazoles (N).

Seven isomeric pairs of 1,2,3-thiadiazole and 1,2,3-triazole derivatives were investigated to reveal their peculiarities in fragmentation and mutual transformations. The structures of these derivatives varied in amine moiety at the fifth position in 1,2,3-thiadiazoles (piperidinyl, pyrrolidinyl, morpholinyl, and amino(N,N-dimethyl)), the corresponding 1,2,3-triazoles, and the sulfonyl substituent (methyl, ethyl, 4-methylphenyl); these are shown in Figure 1. All MS/MS data confirmed by MS^3^ experiments are summarized in fragmentation schemes (Scheme 2, Scheme 3, Scheme 4, Scheme 5, Scheme 8 and Scheme 9).

### 2.1. (+)ESI-MS/MS Study of Compounds ***1a***

**Figure 2:**
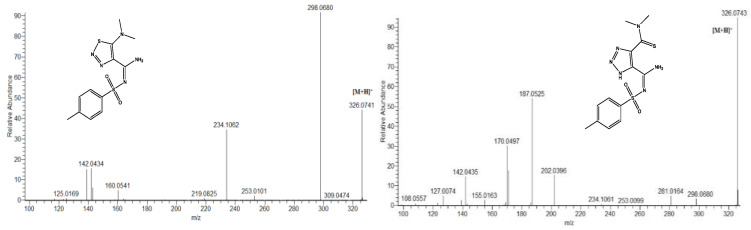
(+)ESI-MS/MS mass spectra of isomeric 1,2,3-thiadiazole **1aS** and 1,2,3-triazole **1aN**.

A number of 4,5-functionalized 1,2,3-thiadiazoles and 1,2,3-triazoles, with alicyclic amine moiety in the fifth position, were studied earlier within our group [22]. Substitution of amine moiety in the fifth position of the 1,2,3-thiadiazole derivative for aliphatic (N,N-dimethylamine) did not significantly influence the fragmentation pattern of protonated 1,2,3-thiadiazole (Figure 2), showing the formation of similar fragment ions as for alicyclic amino-derivatives. Loss of an N_2_ molecule was the main fragmentation process, giving rise to the majority of the observed fragment ions. Unlike the results shown previously [22], bond fission in a sulfonylaryl fragment is possible and may result in charge retention on both sides (*m*/*z* 139, 160, Scheme 2). At some point in the process, the tandem mass spectrum for N,N-dimethylamine-derivative becomes simpler due to the lack of fragment ions which appear due to cleavage in the alicyclic amine moiety.

In the case of **1aN** 1,2,3-triazole (Figure 2), the main fragmentation direction of the protonated molecule is defined by the cleavage in the sulfonamide moiety and the loss of the dimethylamine molecule (Scheme 3). Although the charge retention for the fragmentation induced at a sulfonamide group may take place on both sulfonylaryl (*m*/*z* 155) and heterocyclic fragments, the majority of observed ions possess the structure of 1,2,3-triazole: *m*/*z* 187 ([M+H-ArSO]^+•^), *m*/*z* 171 ([M+H-ArSO_2_]^+•^, *m*/*z* 170 ([M+H-ArSO_2_H]^+^). The structure of *m*/*z* 187 ion ([M+H-ArSO]^+•^) and its analogues was reconsidered in comparison to the one proposed earlier [22] in favor of an aromatic structure, because it provides a better charge delocalization and hence increases the stability of the ion. This type of fragmentation is usually characteristic of sulfones under electron ionization (EI) conditions [30,31], which are described through isomerization of the initial structure due to migration of the radicals from the sulfur atom to one of the oxygen atoms. Similar to what we have seen in previous studies [22], 1,2,3-triazoles do not tend to lose N_2_ molecules. However, fragmentation involving the loss of the amine (*m*/*z* 281—[M+H-C_2_H_7_N]^+^, *m*/*z* 217—[M+H-C_2_H_7_N-SO_2_]^+^) from the thioamide fragment has not earlier been observed in the case of alicyclic derivatives. Additionally, the presence of *m*/*z* 298, 234, and 142 ions in the tandem mass spectrum signifies the rearrangement process of protonated 1,2,3-triazole into the corresponding 1,2,3-thiadiazole. MS^3^ experiments carried out for these ions show complete coincidence of fragment ions with those observed during fragmentation of 1,2,3-thiadiazole.

### 2.2. (+)ESI-MS/MS Study of Compounds of ***1b-g***

Variation in the substituent in the aromatic ring of the sulfonamide fragment was shown earlier [22] to have no significant influence on the fragmentation pathway. Thus, it seems reasonable to investigate the substitution of the aromatic group for an alkyl group in the sulfonamide moiety. A variety of amino/thiocarbamide derivatives of 1,2,3-thiadiazoles and 1,2,3-triazoles with methyl- and ethyl substituents in the sulfonamide group were studied (**1b-g**). To clearly distinguish the effect of the sulfonamide moiety on the fragmentation pattern, the rest of the molecule had the same structure. Fragmentation of the protonated 1,2,3-thiadiazoles **1bS-1gS** was very similar to that observed in the case of the aryl sulfonamide derivatives. All basic fragmentation processes are demonstrated in the example of compound **1eS** (Scheme 4).

It is clear that the majority of observed fragment ions are similar to those found earlier [22]. The main difference between the MS^2^ and MS^3^ results of compounds **1bS-1gS** is a very low abundance of ions undergoing isomerization of the sulfone group with further migration of the neighboring groups (*m*/*z* 212, 200 in Scheme 4). This observation is in full agreement with the peculiarities of fragmentation of sulfone derivatives under EI, where migration of an aryl group is more favorable than that of an alkyl [30,31]. Although the studied protonated 1,2,3-triazoles **1bN-1gN** have demonstrated fragmentation pathways similar to those of sulfonylaryl derivatives under (+)ESI-MS/MS conditions, i.e., the losses of AlkSO, ArSO_2_, and SO_2_ (*m*/*z* 227, 211, and 240, respectively, in Scheme 5), they also showed a wider variety in gas-phase rearrangement processes into the corresponding 1,2,3-thiadiazoles. The common rearrangement of the initial 1,2,3-triazole is observed through the formation of the ion [M+H-N_2_]^+^, which was earlier demonstrated to be characteristic only for 1,2,3-thiadiazoles. This process is followed by further fragmentation resulting in the emergence of ions with *m*/*z* 183 (C_8_H_13_N_3_S), 182 (C_8_H_12_N_3_S), 154 (C_6_H_8_N_3_S), and 150 (C_8_H_12_N_3_) in Scheme 5. The analogous ions were found in MS^3^ spectra of the [M+H-N_2_]^+^ ion of the corresponding 1,2,3-thiadiazoles, supporting the rearrangement hypothesis. However, it appears that the transformation of 1,2,3-triazoles into 1,2,3-thiadiazoles under the (+)ESI-MS/MS conditions may proceed not only through direct isomerization of the initial protonated molecule but also during the fragmentation of ions initially bearing a 1,2,3-triazole structure. The MS^3^ experiment with the [M+H-SO_2_]^+^ ion revealed the presence of the *m*/*z* 183 (C_8_H_13_N_3_S) ion, which has further shown the same set of fragment ions in the MS^4^ experiment as that originating from 1,2,3-thiadiazole (Scheme 5, *m*/*z* 182, 154, 150). The only way to rationalize this observation is by proposing identical structure to the *m*/*z* 183 species arising from the [M+H-SO_2_]^+^ ion. Rearrangement of [M+H-SO_2_]^+^ is likely taking place during the loss of the C_2_H_5_^•^ radical with further elimination of the N_2_ molecule, as the latter is possible only from the 1,2,3-thiadiazole structure. It is apparent that the [M+H-SO_2_]^+^ ion still has a 1,2,3-triazole structure, which is proved by the formation of fragment ions (*m*/*z* 212, 157, and 155 in Scheme 5). Another example of targeted rearrangement during fragmentation is the [M+H-AlkSO_2_]^+^ ion (*m*/*z* 211 in Scheme 5). Fragment ions and corresponding losses—for example, the loss of the NH_3_ molecule observed in the MS^3^ experiment—could be better rationalized by the 1,2,3-thiadiazole structure of the parent ion rather than by the 1,2,3-triazole one. Thus, the introduction of an alkyl moiety next to the sulfonyl group does not significantly change the fragmentation pattern of 1,2,3-triazoles, but at the same time it increases the extent of rearrangement during the (+)ESI-MS/MS experiment.

### 2.3. (+)ESI-MS/MS-IRMPD Spectroscopy Study of Isomeric Structures

**Scheme 6:**
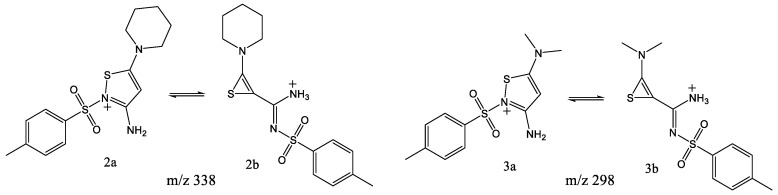
Isomeric structures (2a/2b and 3a/3b) of [M+H-N_2_]^+^ ion originating from 1,2,3-thiadiazole derivatives.

Loss of N_2_ was earlier [22] shown to be the main fragmentation process in CID mass spectra of 1,2,3-thiadiazole derivatives and initially two structures were proposed for the resulting fragment ions—one bearing a thiirene moiety and the other bearing an isothiazole moiety (*m*/*z* 298 and 338; see Scheme 6). ESI-MS/MS alone cannot distinguish between these structures [22], so IRMPD-action ion spectroscopy combined with DFT calculations was applied to identify the correct structure.

IRMPD-action ion spectroscopy, unlike the traditional transmission IR spectroscopy of organic compounds, relies on the fragment yield as a function of excitation wavelength (provided by a tunable IR laser). The extent of fragmentation of the precursor ion (i.e., the fragment yield) depends on its ability to absorb IR photons at a given laser wavelength. This ability is represented by the infrared absorption spectrum of the ion. As the IR spectra of ions are not readily available in reference databases (unlike the IR spectra of neutral organic molecules), they have to be calculated theoretically. This necessitates knowledge of the precursor ion structure. Once the structure is drawn, it is optimized by DFT calculations (or another computational method); its IR absorption spectrum is computed and compared to the experimental action spectroscopy profile. In the case of multiple potential isomers, each of their individual structures and IR spectra must be calculated and compared to the experimental action IR spectrum [24,25].

In calculating the relative energy of thiirene and isothiazole (Scheme 6) structures, the latter was found to be lower in energy by a substantial margin of 259.1 kJ/mol. This is not surprising, given that **2a** has a more stable five-membered thiazole ring and an amino group, as opposed to **2b**, which has a strained three-membered thiirene moiety. IRMPD spectroscopy confirms these theoretical findings.

**Figure 3:**
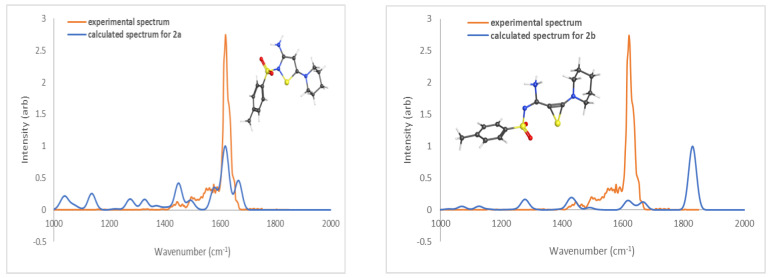
Calculated (blue trace) IR spectra (B3LYP/6-311+g(d,p)) for **2a** (0.0 kJ/mol) and **2b** (259.1 kJ/mol) vs. experimental (orange trace) IRMPD spectrum of the ion *m*/*z* 338.

As can be seen in Figure 3, there is a good match between the experimental IRMPD spectrum (blue trace) and the theoretical IR spectrum of 2a (orange trace). The most prominent absorption band at 1616 cm^−1^ shows a close match to the C-N stretch (between C15 and N22) coupled with C-H wags. The other predicted absorptions, at 1664 cm^−1^ (calculated for NH_2_ scissoring motion) and 1448 cm^−1^ (C=N stretch coupled with N-H wag), are located in areas where some experimental absorption occurs, although with lower intensity. The higher-energy isomeric structure 2b can be rejected because of the absence in the experimental spectrum of the strong absorption band at 1828 cm^−1^ that corresponds to the C=C stretch of the thiirene ring (orange trace).

**Figure 4:**
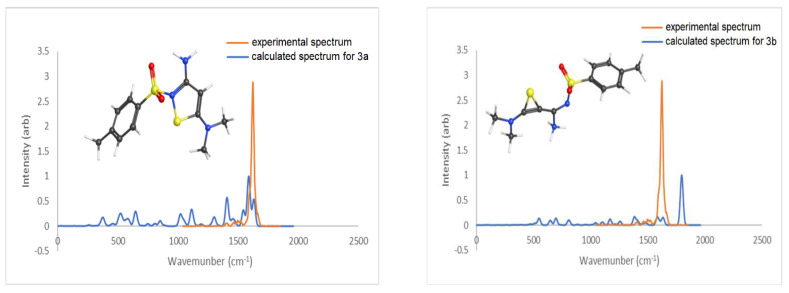
Calculated (blue trace) IR spectra (B3LYP/6-311+g(d,p)) for **3a** (0.0 kJ/mol) and **3b** (264 kJ/mol) vs. experimental (orange trace) IRMPD spectrum of the ion *m*/*z* 298.

Similar to the 2a/2b ions, two potential structures were considered for the *m*/*z* 298 ion, **3a** and **3b**. In this case, the isothiazole structure (**3a**) was also found to be substantially lower in energy, by 264 kJ/mol. This energy difference is close to that determined for the two isomers considered for *m*/*z* 338, as *m*/*z* 298 differs only by the dimethylamine group rather than the piperidinyl moiety. IRMPD ion-spectroscopy findings are also similar and in agreement with theoretical calculations. As shown in Figure 4, there is an acceptable match between the experimental IRMPD spectrum (orange trace) and the theoretical IR spectrum of **3a** (blue trace). The most prominent strong absorption band at 1640 cm^-1^ in the experimental trace matches the absorption location of the two strong bands in the calculated spectrum, the C-N (between C15 and N22) stretch coupled with C-C stretches in the thiazole ring at 1620 cm^−1^ and NH_2_ scissoring mode at 1664 cm^−1^. The higher-energy isomeric structure **3b** can again be rejected because of the absence in the experimental spectrum of the strong absorption band at 1833 cm^−1^ that is calculated for the C=C stretch of the thiirene ring (blue trace).

**Scheme 7:**
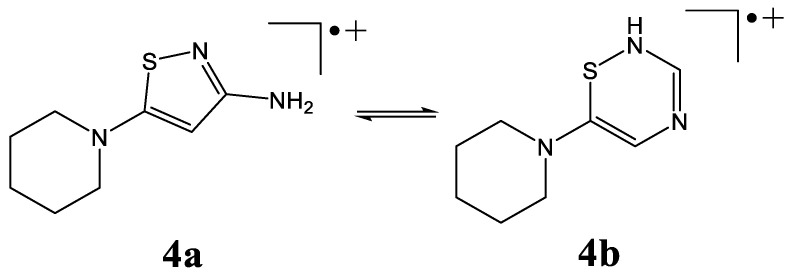
Isomeric structures (**4a** and **4b**) of [M+H-N_2_-RSO_2_]^+^ ion originating from 1,2,3-thiadiazole derivatives.

Another pair of isomeric structures may be proposed for the [M+H-N_2_-RSO_2_]^+^ ion of *m/z* 183 in the case of the piperidinyl derivative (Scheme 7). The first one, **4a**, containing a five-membered thiazole ring and an amino group, was found to be 84.5 kJ/mol more stable than the **4b** structure, which had a six-membered ring containing two nitrogen atoms and one sulfur atom.

**Figure 5:**
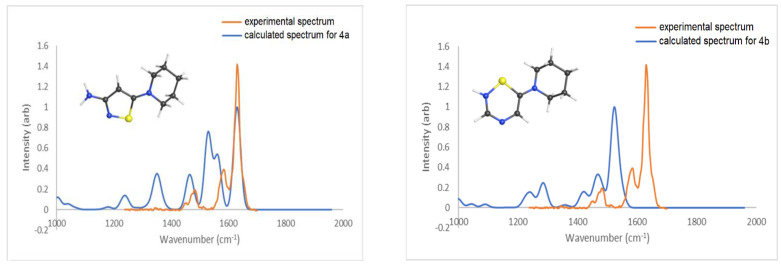
Calculated (blue trace) IR spectra (B3LYP/6-311+g(d,p)) for **4a** (0.0 kJ/mol) and **4b** (84.5 kJ/mol) vs. experimental (orange trace) IRMPD spectrum of the ion *m*/*z* 183.

As shown in Figure 5, the lower-energy isomer displays a reasonable match between the experimental and calculated IR spectra. Most notably, the strong absorption band around 1650 cm^−1^ matches reasonably well to the C-N stretch of the amino group coupled with NH_2_ scissoring motion. The higher energy isomer 4b should have a strong absorption at around 1550 cm^−1^ corresponding to the C-N stretch (between C11 and N4) coupled with C-H wags, but this is absent in the experimental spectrum.

### 2.4. (−)ESI-MS/MS Study of Compounds ***1b-g***

**Figure 6:**
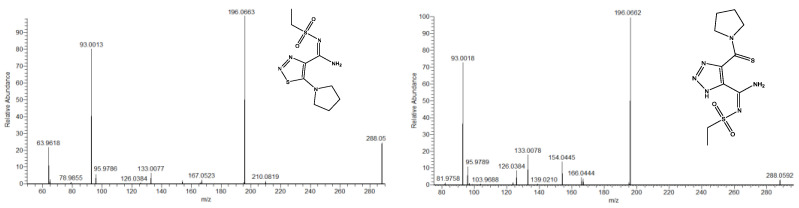
Example of tandem mass spectra of isomeric deprotonated 1,2,3-thiadiazole and 1,2,3-triazole in negative ion mode (compounds **1cS** and **1cN**, respectively).

It was previously demonstrated that the (−)ESI-MS/MS spectra of 1,2,3-triazoles and 1,2,3-thiadiazoles bearing an aromatic substituent on the sulfonamide moiety were very similar [22]. The sulfonylaryl fragment almost completely retained the charge, leaving the rest of the structure invisible in (−)ESI-MS/MS. By changing the aryl moiety for an alkyl one, we expected to observe some fragments revealing the structure of the heterocycle and, possibly, the rearrangement of 1,2,3-thiadiazoles into 1,2,3-triazoles. However, although this time the mass spectra were filled with a larger number of different fragment ions (Figure 6), there were no obvious signs of rearrangement.

The main fragmentation peculiarities in the (−)ESI-CID spectra of compounds **1bS-1gS,** and **1bN-1gN** were summarized and demonstrated with the example of the **1fS** and **1fN** pair, shown in Scheme 8 and Scheme 9. The main fragmentation pathway for both 1,2,3-thiadiazole and 1,2,3-triazole is similar and implies the loss of the sulfonylalkyl moiety (*m*/*z* 304–*m*/*z* 212 in Scheme 8 and Scheme 9). Further fragmentation revealed in the MS^3^ experiment on the *m*/*z* 212 ion, however, shows both the similarities and the differences. Elimination of the C_2_H_4_ and S atom (elementary sulfur), H_2_S, and N_2_ molecules as well as formation of *m*/*z* 142 (C_6_H_8_ONS) and 105 (C_5_H_3_N_3_) ions are among the common processes. As these ions appear in the (−)ESI-CID spectra of both 1,2,3-thiadiazoles and 1,2,3-triazoles, they might signify a possible rearrangement taking place to some extent. At the same time, the structures of the mentioned ions may also be rationalized without any transformation of the central heterocyclic core, i.e., 1,2,3-thiadiazole to 1,2,3-triazole. This thesis agrees with the fact that different ions were recorded in the MS^3^ experiment with the *m*/*z* 212 ion. For instance, the *m*/*z* 212 ion from 1,2,3-thiadiazole forms *m*/*z* 124 (C_4_H_2_N_3_S), while the *m/z* 212 ion from 1,2,3-triazole forms *m*/*z* 184 (C_7_H_10_ON_3_S) and 170 (C_6_H_8_ON_3_S). Hence, we cannot propose an identical structure for the two *m*/*z* 212 ions. It is possible that some part of 1,2,3-thiadiazole is converted to the corresponding 1,2,3-triazole, but there is insufficient evidence to support this hypothesis.

Other fragment ions observed in the MS^2^ spectra are also similar (*m*/*z* 183, 133, 96, 93) for both isomers. Light ions of *m*/*z* 133, 96, 93 most likely result from deep fragmentation processes or charge retention on the sulfonylalkyl fragment, so they could not be used to confirm rearrangement, while the *m*/*z* 183 ion may originate from both thiadiazole or triazole moieties (Scheme 8 and Scheme 9). Therefore, in the case of sulfonylalkyl-1,2,3-thiadiazoles and 1,2,3-triazoles, the observed fragmentation demonstrates all structural features of the original molecule. Transformation of 1,2,3-thiadiazoles to 1,2,3-triazoles under (−)ESI-MS/MS conditions may take place; however, there is no indication of that.

## 3. Materials and Methods

### 3.1. Synthesis of 4,5-Functionalized 1,2,3-Thiadiazoles and 1,2,3-Triazoles

The synthesis of 5-amino-1,2,3-thiadiazol-4-carbimidamides was carried out via reaction of various 2-cyanothioacetamides and sulfonyl azides using aqueous sodium hydroxide according to [12]. The 5-amino-1,2,3-thiadiazol-4-carbimidamides obtained this way were further transformed to 5-sulfonamido-1,2,3-triazole-4-carbothioamides by stirring sodium ethoxide solution into anhydrous ethanol at +23 °C for 1 h.

### 3.2. Mass Spectrometry

ESI-MS/MS experiments were performed using the Orbitrap Elite mass-spectrometer (Thermo Fisher Scientific, Bremen, Germany) equipped with an electrospray ionization source. Acetonitrile solutions of all compounds were analyzed by direct infusion into the ion source through a syringe pump at a flow rate of 10 μL/min. Mass spectra were recorded in both positive and negative modes. Collision-induced dissociation (CID) and higher-energy collisional dissociation (HCD) techniques at a wide range of fragmentation energies were used to obtain the tandem mass spectra. The formulae of all fragment ions were confirmed within 5ppm mass accuracy. Details of the procedure carried out for all samples are described in [22].

### 3.3. Ion Spectroscopy

Gas-phase infrared ion spectroscopy experiments were performed at the FELIX laboratory in Nijmegen, the Netherlands, using an electrospray ionization source on a Bruker amaZon quadrupole ion trap mass spectrometer, modified to provide optical access to the trapped ions [32]. The flow rate of the triazole or thiadiazole sample to the source was 120 μL/hr with a spray voltage of −4500 V and N_2_ as the nebulizer gas. Target fragment ions were generated through ESI-MS/MS of [M+H]^+^ or corresponding precursor ions, mass-isolated in the ion trap, and irradiated with a single infrared laser pulse from the FEL (repetition rate 10 Hz, pulse energies between 80 mJ and 200 mJ). The laser frequency was tuned over the 1000−1850 cm^−1^ range. Mass spectra recorded after irradiation were used to determine the IRMPD yield at each wavelength, which was defined as the ratio of the summed-product ion intensities divided by the total ion intensity. After measuring the intensities of the precursor and fragment ions at a given wavelength of irradiation, the IR frequency was changed in steps of 3 cm^−1^. For each IR frequency, new packets of ions were loaded into the ion trap and irradiated. The intensities of the precursor and product ions were the average of five replicate mass spectra per IR step. The whole process continued across the fingerprint spectral region (1000−1850 cm^−1^). IRMPD spectra were linearly corrected for variations in laser power as a function of IR frequency [33]. The experimental gas-phase IRMPD spectra were then compared to spectra calculated at the density functional theory (DFT) level.

### 3.4. Computational Details

The relative energy of the three couples of fragments with m/z 338, 298 (Scheme 6) and 183 (Scheme 7) was estimated using DFT calculations. The geometry optimization of each of the 6 fragment structures was calculated at the B3LYP/6-311+G(d,p) level of theory using Gaussian 09 [34]. These calculations provided initial clues for the identification of the most thermodynamically stable isomer in each couple of fragments.

Vibrational frequencies calculations were also run at the same level of theory on each of the 6 fragment structures after geometry optimization. This allowed for the prediction of a synthetic IR spectral profile for each of the fragment ions. For gas-phase systems, the theoretical IR spectra can be directly compared with the experimental IRMPD spectral signature (see Section 3.3), providing significant constrains for the assignment of the experimental isomeric structure.

In addition, the computational IR spectra gives information on the specific vibrational modes responsible for each spectral feature. For example, this capability confirmed the absence of the thiirene ring in the experimental isomer for the *m*/*z* 388 and 298 ions (Figure 3 and Figure 4); thus, providing an additional layer to the identification of the experimental structures.

## 4. Conclusions

The effect of amine and sulfonamide moieties on the (+/−)ESI-tandem mass spectra of isomeric 4,5-functionalized 1,2,3-triazoles and 1,2,3-thiadiazoles was examined. Application of accurate mass measurements together with tandem mass spectrometry demonstrated the possibility of gas-phase rearrangement of 1,2,3-triazoles to 1,2,3-thiadiazoles under (+)ESI-MS/MS conditions independent of the nature of substituents, showing the applicability of the designed approach to mimic the reaction in the condensed phase on a larger variety of heterocyclic derivatives. While major fragmentation processes changed a little, there were still some characteristic ions revealing the structure of both isomers. IRMPD spectroscopy enabled us to more unambiguously determine the structures of some important common fragment ions, i.e., revealing the most plausible structures of [M+H-N_2_]^+^ and [M+H-N_2_-RSO_2_]^+^ ions. Due to this additional information, some structures of the fragment ions proposed earlier were reconsidered and modified. Hence, IRMPD spectroscopy provides a deeper understanding of ESI-MS/MS fragmentation mechanisms.

Tandem mass spectra obtained in the negative ion mode were significantly more informative for sulfonylalkyl derivatives than for sulfonylaryl ones. Some common ions were observed in the spectra of both isomers, possibly via the reverse transformation of 1,2,3-thiadiazoles into the corresponding 1,2,3-triazoles. Further studies are needed to explain these observations at the mechanistic level.

## Data Availability

The data presented in this study are available for a limited time on request from the corresponding author.

## Sample Availability

Samples of all the compounds studied are available from the authors.
